# Demoralising Books

**Published:** 1909-07-10

**Authors:** 


### The Hospital

A Journal of

The Medical Sciences and Hospital Administration.

New Series. No. 124, Vol. V. [No. 11[illegible]6, Vol. XLVI.] SATURDAY, JULY 10, 1909.

### Demoralising Books.

At a recent meeting of the Mothers' Union the members pledged themselves to a campaign against demoralising books. This opens up a topic upon which the opinion and advice of the family doctor is nowadays becoming more and more in requisition. The Union's enterprise is no light one if any measure of collective action is seriously contemplated. Of course, within the narrow circle of a family much may be done by judicious selection of literature. But opinion, even among the most righteous and sober members of society, differs so widely in its estimate of what is and is not demoralising to the youthful mind that concerted acton by a large body of individuals is almost inevitably doomed to failure and confusion.

As a writer in the *Times* observes, there are roughly two classes of bad books. One comprises those whose *raison d'être* is the provision of prurient matter, books which make no pretence of any higher aim than an appeal to sensuality. On this class there cannot be two opinions: they are patently, even professedly vicious, and every decent-minded person condemns and deplores them. But there is a far larger class in the estimate of which wide differences of opinion exist, even among persons whose education, breeding, and moral standards seem identical. For views on the subjects which ought and ought not to be dealt with in literature likely to find its way into the hands of the young are as varied as human nature itself. They are determined largely by tradition, by temperament, and by individual susceptibility to suggestions conveyed by books. Thus each man is driven to judge of the effect of a particular book upon others by experience of its effect upon himself, and none but the most self-opinionated can have unlimited confidence in a judgment based upon so insecure a foundation. It is a well-worn maxim among foreign nations that we English, while as frail as the rest of the civilised world, yet cherish a mistaken notion of national superiority because our backslidings are not flagrantly displayed; that, in fact, we share the vices of other nations, and add to them the crowning vice of hypocrisy. No doubt there is some truth in this cynical commentary, but we are insular enough to believe that it is better not to flaunt and glorify vice, even though we permit it, as many think unwisely, to flaunt itself in our streets. Nevertheless, too rigid an exclusion of knowledge on sexual subjects from the educational schedule often over-reaches itself. The very mystery in which it is all enveloped is itself a stimulus to the ready curiosity of youth, which a plain statement of human natural history would satisfy and perhaps deflect into paths of innocent and profitable inquiry. If then we are agreed that it is wise and right to acquaint the young with the formal elementary facts of sex, general agreement seems impossible as to where the line should be drawn between permissible and unpermissible themes. We cannot envy the Mothers' Union the task of achieving unanimity in their censorship of literature. But although any rigid test of suitability for juvenile books seems quite impracticable, one can at least formulate the general lines along which these books may proceed with safety and advantage. Fortunately, the average healthy adolescent cares little for subtleties of character-drawing, but much for thrilling tales packed full of movement, surprises, and adventure. Therefore the writer for the young need not fret himself with analyses and paradoxes of character, but can boldly stamp his puppets good and bad and pass at once to incident. This was the course adopted by the ever-to-be-regretted Henty and Kingston. Artistic literature is thrown away upon the young. At a time when character is still in the making the ideals submitted to it may be crude and overdrawn, provided they are high; and the writer who, for educative purposes, makes virtue always triumphant is lying in a good cause.

In general terms, then, one may say that the more a book is devoted to the exaltation of crude ideals of courage and virtue expressed in conduct under difficult circumstances, the more likely is it to mould character favourably. It is surely better to enter manhood or womanhood with too high than too low a conception of virtue, and better to endure the recurring disillusionments which real life has in store for the optimist, than to leave adolescence a cynic already full-blown. As to the admissibility of sexual references in books for the young we do not pretend to lay down the law, but we believe strongly that such knowledge of sex matters as the young require should be given formally and openly by parents and guardians, or by the family doctor, but not by story-tellers, who may in all, and with the best of intentions, do more harm than good. The intimate post of friend, arbiter, and adviser held by so many physicians in the family councils of their patients, both in times of sickness and of health, is at once a high honour and a great responsibility; and the frequency with which this relationship involves quasi-medical decisions upon the ethical no less than the physiological aspects of sex, is our excuse (if any indeed be necessary) for this ventilation of so homely a subject.

